# Characterization of Functional Effects of Two New Active Fractions Isolated From Scorpion Venom on Neuronal Ca^2+^ Spikes: A Possible Action on Ca^2+^-Dependent Dependent K^+^ Channels

**DOI:** 10.32598/bcn.9.10.352

**Published:** 2019-01-01

**Authors:** Hanieh Tamadon, Zahra Ghasemi, Fatemeh Ghasemi, Narges Hosseinmardi, Hossein Vatanpour, Mahyar Janahmadi

**Affiliations:** 1. Department of Physiology, Neuroscience Research Center, School of Medicine, Shahid Beheshti University of Medical Sciences, Tehran, Iran.; 2. Department of Physiology, School of Medicine, Tarbiat Modares University, Tehran, Iran.; 3. Department of Toxicology and Pharmacology, School of Pharmacy, Shahid Beheshti University of Medical Sciences, Tehran, Iran.

**Keywords:** Scorpion Venom, Intracellular recording, Calcium spike, Buthotus schach

## Abstract

**Introduction:**

It is a long time that natural toxin research is conducted to unlock the medical potential of toxins. Although venoms-toxins cause pathophysiological conditions, they may be effective to treat several diseases. Since toxins including scorpion toxins target voltage-gated ion channels, they may have profound effects on excitable cells. Therefore, elucidating the cellular and electrophysiological impacts of toxins, particularly scorpion toxins would be helpful in future drug development opportunities.

**Methods:**

Intracellular recording was made from F1 cells of Helix aspersa in the presence of calcium Ringer solution in which Na^+^ and K^+^ channels were blocked. Then, the modulation of channel function in the presence of extracellular application of F4 and F6 toxins and kaliotoxin (KTX; 50 nM and 1 μM) was examined by assessing the electrophysiological characteristics of calcium spikes.

**Results:**

The two active toxin fractions, similar to KTX, a known Ca^2+^-activated K^+^ channel blocker, reduced the amplitude of AHP, enhanced the firing frequency of calcium spikes and broadened the duration of Ca^2+^ spikes. Therefore, it might be inferred that these two new fractions induce neuronal hyperexcitability possibly, in part, by blocking calcium-activated potassium channel current. However, this supposition requires further investigation using voltage clamping technique.

**Conclusion:**

These toxin fractions may act as blocker of calcium-activated potassium channels.

## Highlights

- Active fractions isolated from Buthotus schach venom produce hyperexcitability.
- These toxin fractions act similar to Kaliotoxin as a KCa^2+^ channels.
- The two active toxin fractions altered the Ca^2+^ spike parameters.

## Plain Language Summary

Toxins are generally believed to harm human beings, but they may have potential medical applications. Neurons are excitable cells and exhibit action potentials. Neuronal excitability may be altered in diseases. In the present study, the effect of two scorpion venom fractions isolated from *Buthotus schach* were examined on neuronal Ca^2+^ excitability. The findings indicate that application of these toxins reduce neuronal excitability. Therefore, in some neurological diseases (e.g. epilepsy in which hyperexcitability occurs), these venoms may have potential therapeutic use.

## 1. Introduction

Venoms are composed of a large number of bioactive substances, which may have specific effects on the biological systems (Biswas et al., 2012). Although venoms/toxins mainly result in pathophysiological consequences on human, there are several studies that support the potential medicinal properties of natural animal and insect venom neurotoxins including scorpion toxins (Hwang, Kim, & Bae, 2015).

The same target molecules can be affected by many natural toxins in order to control and/or treat several diseases (Mouhat, Jouirou, Mosbah, De Waard, & Sabatier, 2004; Mouhat, Andreotti, Jouirou, & Sabatier, 2008). In this context, ion channels could be common biological targets affected by both diseases and venomous neurotoxins. Functional alterations of many neuronal ion channels in diseases and/or following the exposure to venoms are extensively reported (Mouhat et al., 2004; Possani, Becerril, Delepierre, & Tytgat, 1999; Catterall et al., 2007; Han et al., 2011; Quintero-Hernández, Jiménez-Vargas, Gurrola, Valdivia, & Possani, 2013).

Ion channels have different fundamental regulatory roles in neuronal excitability; therefore they could be considered as potential therapeutic and /or preventive targets. Heterogeneity in the expression of ion channel proteins shapes action potential characteristics and discharge firing pattern (Bean, 2007; Palacio et al., 2010); therefore, analysis of the impact of natural toxins on the shape of action potential or cell excitability would be beneficial in the early stages of drug development (Mohan, Molnar, & Hickman, 2006; Akanda, Molnar, Stancescu, & Hickman, 2009). Among them, voltage-gated Na^+^, Ca^2+^, and K^+^ channels are important therapeutic candidates which can be modulated by various neurotoxins including scorpion toxins (Batista et al., 2002; Zuo & Ji 2004; Quintero-Hernández et al., 2013; He et al., 2016).

Voltage-gated K^+^ channels are crucial to regulate the neuronal excitability, through contribution to the repolarization following a potential action. Their blockade results in neuronal hyperexcitability by reducing the membrane hyperpolarization potential. Several types of potassium channels, including Ca^2+^-activated K^+^ channels are reported to exist in different neuronal cell types (Humphries & Dart, 2015).Therefore, characterizing the functional effects of new scorpion toxin fractions may affect the potassium channel functions, particularly KCa^2+^ is important and could be a promising candidate as a KCa^2+^ channel blocker to treat diseases (Devaux, 2010; Bittner & Meuth, 2013; Ehling, Bittner, Budde, Wiendl, & Meuth, 2011; Martin et al., 2017). Calcium-activated K^+^ channels contribute to the regulation of vesicular release of neurotransmitters (Lee & Cui, 2010).

Kaliotoxin (KTX), an Androctonus mauretanicus mauretanicus peptidyl neurotoxin, is reported to block neuronal maxi Ca^2+^-activated K^+^ channels in snail neurons (Crest et al., 1992). KTX is widely used to treat experimental autoimmune encephalomyelitis (Beeton et al., 2001) and inflammatory lesions of periodontal disease (Valverde, Kawai, & Taubman, 2004). It was also used to facilitate cognitive processes such as learning (Kourrich, Mourre, & Soumireu Mourat, 2001); therefore it was suggested that KTX-sensitive potassium channels contribute to the repolarization of the presynaptic action potential of hippocampal inhibitory neurons and thereby induce facilitation of synaptic transmission (Martin-Eauclaire, & Bougis, 2012).

In the current study, the electrophysiological consequences of two new fractions (F4 and F6) isolated from Buthotus schach scorpion venom were investigated on the properties of neuronal Ca^2+^ spikes. The scorpion B. schach, which belongs to the Buthidae family, is widely found in the western and tropical areas of Iran. In the authors` previous report, the effect of these two new fractions was investigated on the release of Ach in neuromuscular junctions (Vatanpour, Ahmadi, Zare-Mirakabadi, & Jalali, 2012), where these two fractions transiently increased the amplitude of muscle twitch associated with a huge contracture and then followed by muscle paralysis in chick and mice (Vatanpour et al., 2012). It was also shown that application of both fractions affected the Na^+^ action potential waveform of F1 neurons of Helix aspersa (Tamaddon, Ghasemi, Vatanpour, & Janahmadi, 2014).

In the current study, it was attempted to demonstrate the functional effects of the two fractions on the electrophysiological properties of Ca^2+^ spikes in F1 neurons of snail neurons. Findings of the present account extend the findings presented in the authors` previous work (Tamaddon et al., 2014) by providing additional details regarding the effects of the two active toxin fractions on Ca^2+^-dependent neuronal excitability.

## 2. Methods

All recordings were performed on the soma membrane of F1 neuronal cells, located on the right parietal of suboesophageal ganglia of H. aspersa (Iranian garden snail). The ganglionic mass was dissected out and then pinned on the bottom of the recording chamber covered by Sylgard184 (Dow Corning Midland, MI, USA). Thereafter, the superficial connective tissue overlying the ganglia was gently removed using fine forceps.F1 neurons were then visualized under stereomicroscope (Nikon, Japan) by their location and size within the right parietal ganglion. Intracellular recordings were performed in the presence of calcium Ringer solution in which Na^+^ was replaced by Tetraethylammonium (TEA) chloride and voltage-gated K^+^ channel current was blocked by bath application of 4-aminopyridine (4-AP) and TEA.

The Ca^2+^ bathing solution contained: 80 mM TEA, 4 mM KCl, 10 mM CaCl2, 5 mM MgCl2, 10 mM glucose, and 5 mM HEPES (4-(2-hydroxyethyl)-1-piperazineethanesulfonic acid). In order to examine the electrophysiological consequences of neuronal exposure to the two toxin fractions or KTX, as a standard scorpion toxin, on Ca^2+^ excitability, two doses (50 nM and 1 μM) of the toxin solution were applied on the basis of respective literature and prior works in the laboratory (Tamadon et al., 2014). The six toxin fractions were isolated and purified (Vatanpour et al., 2012; Aboutorabi, Naderi, Gholamipourbadie, Zolfagharian, & Vatanpour, 2016) and also the two fractions that had action mostly prejunctionally on Ach release from the neuromuscular junctions of chicks and mice (Vatanpour et al., 2012).

Intracellular recording technique was employed under current-clamp condition using an Axoclamp 2B amplifier (Axon Instruments, Foster City, CA, USA). An Ag/AgCl electrode within an agar bridge (4% agar in snail Ringer) was used as a reference or ground electrode. Spontaneous Ca^2+^ spikes were recorded in the presence or absence of either toxin fractions (F4 or F6) or KTX. Voltage signals were filtered at 10 kHz and digitized at 20 kHz using a 16 bit A/D converter (ADInstrument Pty Ltd., Sydney, Australia) and stored on a computer for further offline analysis using Lab Chart pro7 and Excel software.

### 2.1 Statistical analysis

Results were reported as Mean±SEM with ‘n’ being the number of cells on which the recording was performed. Data were subjected to statistical analysis with Graph-Pad Prism 6 software, using unpaired Student t-test or one-way ANOVA followed by Tukey test as the post hoc analysis. P≤0.05 was considered significant.

## 3. Results

**Figure 1.**
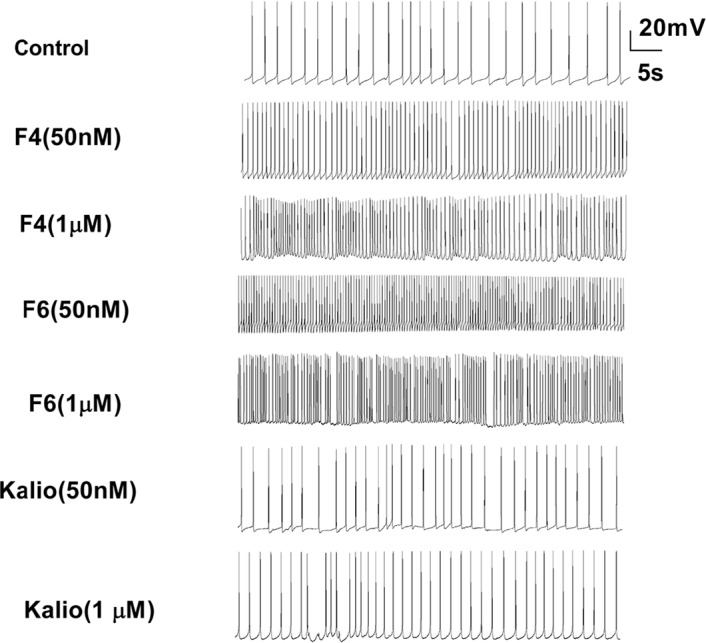
Effects of two different concentrations of F4 and F6 fractions isolated from Buthotus schach scorpion venom and KTX on the spontaneous calcium spike firing Extracellular application of either F4 and F6 or KTX resulted in the neuronal hyperexcitability.

**Figure 2.**
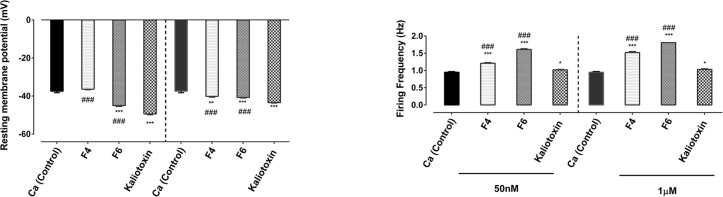
Effect of two neurotoxins and KTX on the AHP amplitude and the half-width of Ca^2+^ spike (A) Application of either the two active fractions, F4 and F6, or KTX caused a significant reduction in the AHP amplitude; (B) Neuronal exposure to all applied neurotoxins led to a significant spike broadening, except KTX 50 nM, which reduced the duration of calcium spike. (C) Superimposed Ca^2+^ spikes in the control condition and after application of the two active toxin fractions and KTX. (B) *Indicates significant difference between the control group and all neurotoxins treated groups (P<0.001, P<0.01); # shows significant difference between KTX and the two active toxin fractions (P<0.05, P<0.01, P<0.001).

**Figure 3.**
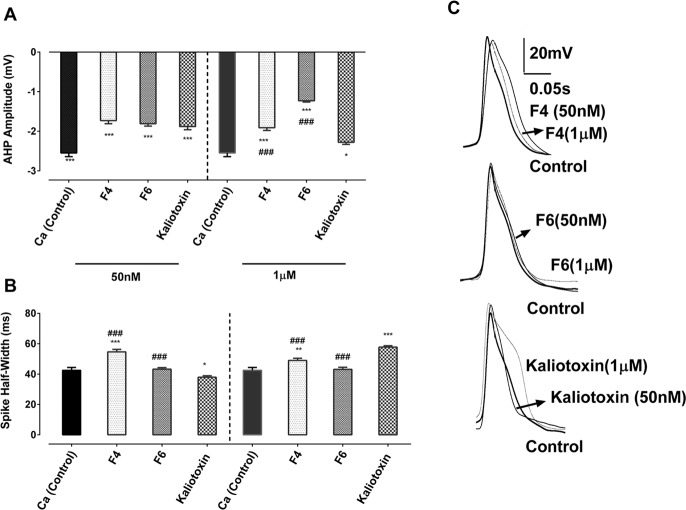
Effect of F4, F6, and KTX on the F1 cell electrophysiological properties The impact of toxins on the resting membrane potential (A), action potential duration (B), and spike frequency (C); * indicates significant difference between the control group and all neurotoxins treated groups (P<0.05, P<0.01, P<0.001); # shows significant difference between KTX and the two active toxin fractions (P<0.05, P<0.01, P<0.001)

Calcium channel modulators can regulate membrane excitability in many neurons in part by changing the AHP amplitude. Thus, the Ca^2+^spikes were recorded from the soma after blockade of the inward Na^+^ channel and outward voltage-gated K^+^ channels (Figure 1). Under this condition, the mean of neuronal Resting Membrane Potential (RMP) was −37.43±0.8 mV (Figure 2A), the spike firing frequency was 0.95±0.02 Hz (Figure 2B), the amplitude of After Hyperpolarization Potential (AHP) was −2.55±0.09 mV and the half-width of Ca^2+^ spike was 42.52±1.84 ms (Figures 3A, C).

When F1 neurons were exposed to Ca^2+^ Ringer containing F4 fraction at a concentration of 50 nM a slight depolarization in occurred the membrane voltage (−36.38±0.31 mV), but at 1 μM a shift occurred in the membrane potential towards more hyperpolarized voltages (−40.14±0.46 mV, P≤0.01; Figure 2A). The Ca^2+^ spike frequency significantly increased in response to an exposure to both concentrations of F4 toxin fraction (1.21±0.02 Hz, P≤0.001 and 1.52±0.03 Hz, P≤0.001; Figure 2B). Moreover, application of Ca^2+^ Ringer solution containing F4 fraction led to a significant reduction in the amplitude of AHP (−1.73±0.08 mV, P<0.001 and −1.91±0.07 mV, P<0.001; Figure 3A).

The recorded Ca^2+^ spikes significantly broadened when cells were exposed to both concentrations of F4 toxin fraction (54.63±1.67 ms, P≤0.001 and 48.95±1.49 ms, P≤0.01; Figure 3B). Thereafter, the effect of F6 fraction was examined on the electrophysiological properties of Ca^2+^ spikes in a separate set of experiments.

Extracellular application of the F6 fraction at concentrations of either 50 nM or 1μ M significantly shifted the RMP to the hyperpolarized potential (−45.01±0.46 mV, P≤0.001 and −40.74±0.27 mV, P≤0.001, Figure 2A, respectively). In addition, the amplitude of AHP significantly decreased when cells were exposed to both doses of F6 toxin fraction (−1.81±0.06 mV, P<0.001 and −1.23±0.03 mV, P<0.001, Figure 3A, respectively) and this led to a significant increase in the spike firing frequency (1.61±0.02 Hz, P≤0.001 and 1.81±0.02 Hz, P≤0.001 in the presence of 50 nM and 1 μM, Figure 2B, respectively). Application of F6 fraction was also associated with a slight insignificant prolongation of Ca^2+^ spike duration both at a lower dose (43.27±1.01 ms) and higher dose (43.13±1.33 ms, Figure 3B).

### 3.1. The electrophysiological consequences of KTX exposure on the Ca^2+^ spikes

Following the application of KTX, a known scorpion neurotoxin to block KCa current, the RMP shifted towards hyperpolarization potential either in the presence of 50 nM (−49.41±0.39 mV, P≤0.001) or 1 μM concentration of neurotoxin (−43.43±0.26 mV, P≤0.001, Figure 2A). In addition, exposure to KTX significantly dampened the AHP amplitude and an increase in the firing rate (1.02±0.01 Hz, P≤0.05 and 1.03±0.02 Hz, P≤0.05, Figure 3A, respectively). However, application of KTX had dose dependently opposite effects on the duration of Ca^2+^ spike. At low concentration, KTX exposure led to a significant spike prolongation (37.91±0.99 ms, P≤0.05), but at high concentration resulted in shortening the spike (57.75±0.92 ms, P≤0.001; Figure 3B).

### 3.2 Comparison of the effects of KTX and B. schach scorpion venom fractions of F4 and F6 on Ca^2+^ spikes

Comparing the action of potential electrophysiological parameters measured in the presence of KTX and the two active fractions demonstrated that all neurotoxin treatments had the same effects on the measured variables. Therefore, the RMP became more hyperpolarized. In addition, although all applied neurotoxins caused a decrease in AHP amplitude, they exerted an increasing effect on the spike frequency.

## 4. Discussion

The current study attempted to determine the electro-physiological consequences of exposure to the two active toxin fractions isolated from B. schach venom on the Ca^2+^-dependent neuronal excitability. To this end, the ionic conditions were manipulated by replacing tetraethyl ammonium hydrochloride for sodium chloride and by adding 4-AP (5 mM) to block IA channel current. Under this condition, F1 neurones generated overshooting Ca^2+^-dependent spikes. Then, the Ca^2+^ spike parameters, including RMP, AHP amplitude, spike duration, and firing frequency were measured and compared in the presence of the two active fractions with the ones obtained in the presence of KTX, as a known scorpion neurotoxin particularly acting on KCa channel.

Natural toxins are widely used as tools to identify the new biomedical molecules and pathways and also as experimental probes for membrane structures comprising their targets. In addition, the natural toxins can be turned and evolved into life-saving drugs and powerful medications. Therefore, identifying and characterising the impact of the new toxin fractions at the cellular level may be helpful to treat the diseases and develop new drugs. Here, it was investigated whether exposure to the two new scorpion toxin fractions may affect the Ca^2+^-based excitability in the F1 neuron in H. aspersa.

Several studies propose that transient and delayed rectifier K^+^ outward currents (Thompson, 1977; Solntseva, 1995; Bal, Janahmadi, Green, & Sanders, 2000; Bal, Janahmadi, Green, & Sanders, 2001; Sakakibara et al., 2005; Janahmadi, Farajnia, Vatanparast, Abbasipour, & Kamalinejad, 2008), and Ca^2+^ activated K^+^ channels (Hermann & Erxleben, 1987; Gola, Ducreux, & Chagneux, 1990; Crest & Gola, 1993) are responsible for generating AHP following action potential in snail neurons. The authors` previous work demonstrated that functional blockade of Ca^2+^ activated potassium channels, increased the frequency of Ca^2+^ spikes by eliminating the AHP, which follows action potential (Vatanparast, Janahmadi, & Asgari, 2006; Janahmadi et al., 2008). Therefore, increasing the effect of the two new active fractions and KTX on the firing frequency could be possible due to the inhibition of KCa channels.

There are several evidences reporting the effect of KTX on either voltage-gated or calcium-activated potassium channels including Kv1.3 and BK channels, respectively (Lange, Paris, & Celerier, 1992; Crest, Gola, 1993; Zachariae, Kaltoft, & Thestrup-Pedersen, 1992; Aiyar, Rizzi, Gutman, & Chandy, 1996). The blocking effect of KTX on calcium-activated potassium channels was reported by Crest et al. (1992). The function of these channels is a key link between the rise in intracellular free Ca^2+^ and neuronal excitability by affecting the amplitude of AHP and firing frequency (MacDonald, Ruth, Knaus, & Shipston, 2006; Lin, Hatcher, Chen, Wurster, & Cheng, 2010). There are also other reports indicating that exposure to scorpion venom peptides cause the enhancement of neuronal excitability by suppressing the AHP (Ishii et al., 1997; Juhng et al., 1999; Pedarzani et al., 2002). In many neurons, Ca^2+^ entry through activation of Ca^2+^ leads to opening the Ca^2+^ dependent potassium channels and thereby regulates cell excitability (Lancaster, Adams, 1986; Sah & Faber., 2002; Janahmadi et al., 2008; Duménie, Fourcaud-Trocmé, Garcia, & Kuczewski, 2015). In the present study, in common with KTX the two new active scorpion toxin fractions enhanced firing frequency by reducing the amplitude of AHP (Haghdoost-Yazdi, Janahmadi, & Behzadi, 2008). However, the further voltage-clamp analysis is needed to address this issue.

Another finding of the present work was hyperpolarization of the membrane potential following the treatment of scorpion envenoming. Although there are several reports in the literature showing the involvement of Ca^2+^-activated K^+^ channels to generate AHP, which thereby contribute to the repolarization phase and the duration of action potential (Storm, 1987; Liu et al., 2014), not resting membrane potential, blockade of these channels by scorpion toxins caused membrane hyperpolarization. It is hard to provide a decisive causative mechanism for this effect, but it can be hypothesized that blockade of KCa channels by scorpion toxins causes less K^+^ efflux and thereby leads to the accumulation of more positive ions inside the cell. This, in turn, may increase the Na^+^-K^+^ pump activity leading to membrane hyperpolarization.

Neuronal exposure to either of the two active fractions isolated from B. schach or KTX resulted in spike broadening. Since in the present study voltage-gated sodium and potassium channels were blocked, one possible explanation for the alteration in the spike duration could be changes in the balance between inward Ca^2+^ current and outward KCa current. Particularly, possible inhibitory effect of applied neurotoxin could be more effective on the membrane repolarization and the duration of action potential (Ma & Koester, 1996; Faber & Sah, 2003; Battonyai, Krajcs, Serfőző, Kiss, & Elekes, 2014). It is very well documented that H. aspersa neurons possess many types of ion channels including voltage and Ca^2+^ dependent K^+^ channels. Ca^2+^-activated K^+^ channels are divided into three types based on the conductance: big, intermediate, and small conductance KCa channels. The first group consisted of two subtypes, including one sensitive to intracellular Ca^2+^ concentration (BKCa) and one sensitive to the scorpion toxin, charybdotoxin (HLK3 channels). The second type included two kinds of SK channels: SK2 and SK3. BKCa channels activation is involved in action potential repolarization, while SKCa channels contribute to underlie the AHP (Sah, 1996).

In conclusion, findings of the present investigation suggested that the two new scorpion toxin fractions isolated from B. schach venom, similar to the known scorpion neurotoxin, KTX, caused hyperexcitability, possibly by blocking calcium-activated potassium channel current, although further voltage-clamp investigations are needed to explore the properties of ion channels affected by examined venom.

## Ethical Considerations

### Compliance with ethical guidelines

All experiments were approved by the Ethics Committee of Shahid Beheshti University of Medical Sciences.
